# Dengue-like illness surveillance: a two-year longitudinal survey in suburban and rural communities in the Lao People's Democratic Republic and in Thailand

**DOI:** 10.5365/wpsar.2017.8.4.001

**Published:** 2019-02-19

**Authors:** Nanthasane Vannavong, Razak Seidu, Thor Axel Stenström, Nsa Dada, Hans Jørgen Overgaard

**Affiliations:** a Faculty of Science and Technology, Norwegian University of Life Sciences, Ås, Norway.; b Champasak Provincial Health Office, Pakse, Lao People’s Democratic Republic.; c Water and Environmental Engineering Group, Institute for Marine Operations and Civil Engineering, Norwegian University of Science and Technology, Ålesund, Norway.; d SARChl Chair, Institute for Water and Waste Water Technology, Durban University of Technology, Durban, South Africa.

## Abstract

**Objective:**

This study aimed to determine the incidences of dengue-like illness (DLI), dengue virus (DENV) infection, and serotypes and to identify socio-demographical and entomological risk factors of DLI in selected suburban and rural communities in the Lao People's Democratic Republic and in Thailand.

**Methods:**

A two-year longitudinal study was conducted in four villages during the inter-epidemic period between 2011 and 2013. Entomological surveys, semi-structured interviews of household heads and observations were conducted. Occurrences of DLI were recorded weekly using the World Health Organization’s dengue definition along with blood samples; results were compared with national surveillance dengue data. Risk factors of DLI were assessed using logistic regression.

**Results:**

Among the 2007 people in the study, 83 DLI cases were reported: 69 in suburban Lao People's Democratic Republic, 11 in rural Thailand, three in rural Lao People's Democratic Republic and none in suburban Thailand. Four were confirmed DENV: two from suburban Lao People's Democratic Republic (both DENV-1) and two from rural Thailand (both DENV-2). Although the number of detected DLIs during the study period was low, DLI incidence was higher in the study compared to the dengue surveillance data in both countries. DLI in suburban Lao People's Democratic Republic was associated with age and occupation, but not with the number of pupae per person.

**Discussion:**

This study highlights the importance of continuous clinical and vector surveillance for dengue to improve early detection of dengue and other mosquito-borne diseases in the region.

## Objective

Dengue is a mosquito-borne viral infection prevalent throughout the tropics and subtropics. In South-East Asia, one of the largest outbreaks ever recorded occurred in 2010, (*1*–*3*) during which 22 929 cases and 46 deaths were recorded in the Lao People's Democratic Republic, (*2*) and 116 947 cases and 139 deaths in Thailand. (*4*) The incidence in the Lao People's Democratic Republic was 367 cases per 100 000 persons, (*1*) and in Thailand, 177 cases per 100 000 persons—higher than recorded in neighbouring Viet Nam (150 cases per 100 000 persons) or Cambodia (93 cases per 100 000 persons). (*2*) The most recent outbreak in Thailand occurred in 2015 with 144 952 cases and 148 deaths; (*5*) it was the most prevalent circulating strains of dengue virus (DENV) were DENV-4 (33.1%) and DENV-3 (32.6%). (*6*) In the Lao People's Democratic Republic, the most recent large outbreak occurred 2013, with 15 out of 17 provinces reporting dengue at epidemic levels, causing 95 deaths from a total of 44 171 cases; (*7*) DENV-3 and DENV-2 were the most common serotypes. (*8*)

In the Lao People's Democratic Republic and in Thailand, the number of cases peak during the rainy season, generally between May and October. (*1*, *4*) Dengue vector control in affected settings mainly relies on integrated vector management as recommended by the World Health Organization (WHO). (*9*) A widely occurring challenge for effective mosquito control using the larvicide temephos is the widespread belief that it is harmful due to its smell. (*10*) Another challenge is insecticide resistance in *Aedes aegypti*, the main dengue vector, (*11*) which has been identified in Thailand (*11*) and the Lao People's Democratic Republic. (*12*) A review of space spraying for the control of adult mosquitoes revealed that this intervention was unsustainable and did not lead to a reduction in dengue incidence. (*13*) A dengue vaccine is approved for public use in Thailand; however, those without a history of DENV infection before vaccination have been found to have a risk of developing severe disease. (*14*)

Patients with dengue-like illnesses (DLI) have acute febrile illnesses (AFI) with similar clinical manifestations to dengue (*3*) but without laboratory confirmation of dengue infection. DLI can be defined even in settings without laboratory facilities or rapid diagnosis test kits for confirming dengue infection. (*9*, *15*) An etiological study done in the southern Lao People's Democratic Republic in 2003–2004 found that 30% (69/229) of patients presenting with non-malarial febrile illnesses during inter-epidemic periods of dengue had dengue infections confirmed by enzyme-linked immunosorbent assay (ELISA). (*16*) In Thailand, dengue was the third leading cause of AFI in rural areas. (*17*)

Active surveillance of dengue and DENV serotypes in non-outbreak settings is rarely conducted in the Lao People's Democratic Republic or in Thailand. Identifying DENV infection is necessary to reduce the dengue burden by improving the early response and implementing control measures. The aims of this study were to assess the incidence and risk factors of DLI and to identify dengue infections in relation to socio-demographic characteristics and mosquito pupal indices in selected study sites of both countries during an inter-epidemic period.

## Methods

### Study areas and design

**Fig. 1:**
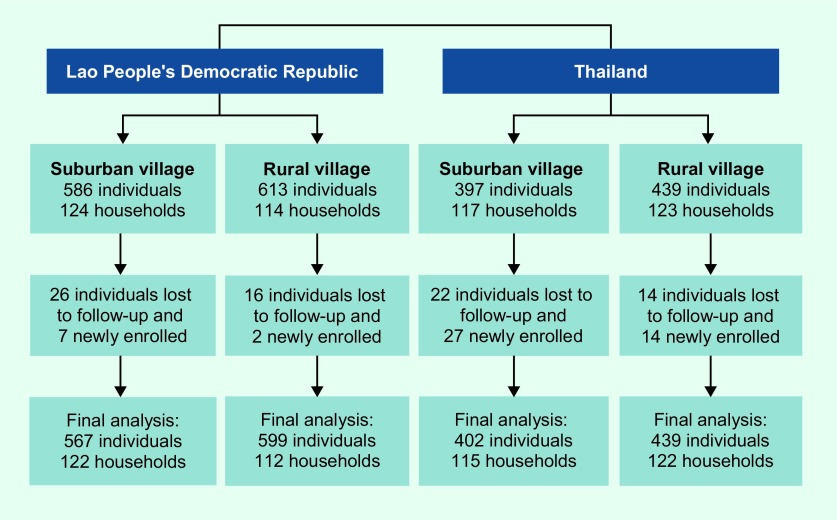
Number of participants and households included in the cross-sectional survey carried out during two years of follow-up from 2011 to 2012 in four villages in the Lao People's Democratic Republic and in Thailand

A two-year longitudinal study with active case detection was conducted in Salavan province, southern Lao People's Democratic Republic and in Khon Kaen province, northeastern Thailand. One suburban and one rural village were selected in each country (**Fig. 1**). The two villages in the Lao People's Democratic Republic and the two villages in Thailand are located six and nine kilometres apart, respectively. These villages were selected based on previously described criteria. (*18*) The study was conducted in both dry and wet seasons within the time period of March 2011 to April 2013 with slight deviations of the start and end dates between the sites.

### Participants

The total number of households in each village was 215 in suburban and 130 in rural Lao People's Democratic Republic, and 272 in suburban and 139 in rural Thailand. Rural Lao People's Democratic Republic had the lowest number of households (130), and we chose this number as the sample size across all selected villages. For each of the other three villages with more than 130 households, we systematically sampled households by first identifying a random house and then selecting additional houses based on a fixed interval derived by dividing the total number of households by 130. All individuals residing in the selected households were included. **Fig. 1** shows the total number of households and people included. During the course of the study, individuals were lost to follow-up because they went to study elsewhere, moved out after marriage or died; households were lost to follow-up due to families moving and settling in other villages or choosing to leave the study. Migrants and newborn children were included as newly enrolled participants. The final number of individuals included in the analysis was 2007.

#### Identification of dengue-like illnesses

Each of the selected households was visited weekly by trained village health volunteers. DLI was defined using the WHO dengue definition, i.e. presence of AFI for 2–7 days with two or more nonspecific symptoms such as headache, retro-orbital pain, myalgia, arthralgia, rash, and haemorrhagic manifestations. (*19*) We used an individual questionnaire to obtain case information (**Table 1**) from patients or guardians of patients less than 15 years old.

**Table 1:** General information of individuals, households, mosquito infestation and disease information (dengue-like illness (DLI) and dengue) in suburban and rural villages in the Lao People's Democratic Republic and in Thailand (percentages in parentheses)

-		Lao People's Democratic Republic		Thailand	
-		Suburban	Rural	Suburban	Rural
**Individuals**					
**No. of individuals**	-	**567**	**599**	**402**	**439**
Age group (in years)	0–5	76 (13.4)	57 (9.5)	22 (5.5)	29 (6.6)
	> 5–10	55 (9.7)	68 (11.3)	25 (6.2)	24 (5.5)
	> 10–15	79 (14.0)	78 (13.0)	39 (9.7)	21 (4.8)
	> 15–20	67 (11.8)	65 (10.9)	26 (6.5)	34 (7.7)
	> 20–25	37 (6.5)	52 (8.7)	19 (4.7)	11 (2.5)
	> 25–30	53 (9.4)	48 (8.0)	21 (5.2)	13 (2.9)
	> 30–35	41 (7.2)	39 (6.5)	17 (4.2)	31 (7.1)
	> 35–40	30 (5.3)	44 (7.4)	16 (4.0)	26 (5.9)
	> 40	129 (22.7)	148 (24.7)	217 (54.0)	250 (56.9)
Mean age (in years)	-	25.7	28.1	40.2	41.6
Gender	Male	274 (48.3)	305 (50.9)	188 (46.8)	221 (50.3)
	Female	293 (51.7)	294 (49.1)	214 (53.2)	218 (49.7)
Occupation	Agriculture	178 (31.4)	392 (65.4)	50 (12.4)	284 (64.7)
	Service	93 (16.4)	19 (3.2)	27 (6.7)	14 (3.2)
	Commerce	33 (5.8)	12 (2.0)	80 (20.0)	8 (1.8)
	Unemployed	8 (1.4)	3 (0.5)	53 (13.2)	9 (2.1)
	Student	146 (25.8)	102 (17.0)	89 (22.1)	62 (14.1)
	Other*	109 (19.2)	71 (11.9)	103 (25.6)	62 (14.1)
**Households**					
**Total households per village**	-	**215**	**130**	**272**	**139**
**No. of selected households**	-	**122**	**112**	**115**	**122**
Room occupancy rate	> 2.5 persons/room	61 (50.0)	43 (38.4)	21 (18.3)	32 (26.2)
	≤ 2.5 persons/room	61 (50.0)	69 (61.6)	94 (81.7)	90 (73.8)
Wealth status	Poor	38 (31.1)	91 (81.2)	5 (4.3)	19 (15.6)
	Intermediate	51 (41.8)	15 (13.4)	34 (29.6)	66 (54.1)
	Rich	33 (27.1)	6 (5.4)	76 (66.1)	37 (30.3)
Housing material	Cement	24 (19.7)	1 (0.9)	23 (20.0)	16 (13.1)
	Cement-wood	48 (39.3)	16 (14.3)	80 (69.6)	74 (60.7)
	Wood	50 (41.0)	95 (84.8)	12 (10.4)	32 (26.2)
***Aedes aegypti* pupal indices†**	-	**No. [min-max]**	**No. [min-max]**	**No. [min-max]**	**No. [min-max]**
No. of pupae in all positive containers	-	903	558	1005	1005
No. of pupae per household	-	7.5 [0–102]	5.0 [0–231]	8.7 [0–184]	8.4 [0–153]
No. of pupae per person	-	1.6 [0–22.5]	0.9 [0–57.8]	2.5 [0–92]	2.3 [0–76.5]
**DLI case information**					
**No. of DLI cases**	-	**69**	**3**	**0**	**11**
DLI cases confirmed to be dengue	-	2	0	0	2
DENV serotypes	-	DENV-1	-	-	DENV-2
Signs and symptoms	Fever	69 (100.0)	3 (100.0)	-	11 (100.0)
	Headache	55 (79.7)	2 (66.7)	-	9 (81.8)
	Orbital pain	23 (33.3)	1 (33.3)	-	7 (63.6)
	Joint pain	38 (55.1)	1 (33.3)	-	9 (81.8)
	Rash	6 (0.9)	0	-	0
	Bleeding	0	0	-	0
Age group (in years)	0–5	4 (5.8)	0	-	0
	> 5–10	7 (10.1)	0	-	1 (9.1)
	> 10–15	12 (17.4)	0	-	1 (9.1)
	> 15–20	12 (17.4)	1 (33.3)	-	1 (9.1)
	> 20–25	6 (8.7)	0	-	0
	> 25–30	6 (8.7)	0	-	0
	> 30–35	6 (8.7)	0	-	0
	> 35–40	8 (11.6)	0	-	2 (18.2)
	> 40	8 (11.6)	2 (66.7)	-	6 (54.5)
Gender	Male	31 (44.9)	0	-	2 (18.2)
	Female	38 (55.1)	3 (100.0)	-	9 (81.8)
Mean number of days staying in village during the week before DLI detection	-	6.6	6.3	-	6.6
Sleeping during the day	No	28 (40.6)	0	-	4 (36.4)
	Yes	41 (59.4)	3 (100.0)	-	7 (63.6)
Dengue protection methods	Mosquito net	69 (100.0)	3 (100.0)	-	8 (72.7)
	Bug zapper	11 (15.9)	1 (33.3)	-	0
	Mosquito coil	4 (5.8)	0	-	3 (27.3)
	Insecticide spray	3 (4.4)	0	-	3 (27.3)
	Repellent	2 (2.9)	0	-	1 (9.1)
	Other	5 (7.3)	0	-	4 (36.4)

† ***Aedes aegypti*** pupal indices applied from Dada et al. (2013). (*18*) Number of ***Aedes aegypti*** pupae was not available in one household of suburban Lao People's Democratic Republic and in two households of rural Thailand

*Other: retired and children, Min-max: minimum-maximum

#### Confirmation of dengue cases

From each identified DLI case, we took a blood sample by finger prick and blotting onto two pieces of filter paper (Blood Sampling Paper, NOBUTO, Chemoscience (Thailand) Co., Ltd), according to the manufacturer’s instructions. After the blood was absorbed, the paper was dried at room temperature for 1–2 hours and thereafter sealed in sterile bags (Whirl-Pak Bags, Chemoscience (Thailand) Co., Ltd). The samples were stored at −20 °C until transport. All samples were periodically brought to Thailand where they were analysed by real-time polymerase chain reaction (PCR) for DENV RNA detection and serotyping using previously described techniques. (*20*) We obtained secondary data reported during 2010–2013 from the national surveillance system in both countries to compare with data from our study.

#### Socio-demographic characteristics and entomological survey

Household information was obtained from household heads using a semi-structured household questionnaire. Data collected are displayed in **Table 1**. The entomological survey was conducted once per household in 2011 from the beginning of March to the beginning of June. In suburban and rural Thailand, the survey was conducted in March–April 2011, while for the Lao People's Democratic Republic villages, the survey was done in May–June 2011. All household water storage containers were examined for *Ae. aegypti* pupae, and the number of pupae present were counted and recorded. Pupae were identified to species using a dissecting microscope and illustrated keys as described elsewhere. (*18*)

#### Data analysis

Descriptive analysis of socio-demographic characteristics and entomological data was conducted for each study village. Room occupancy rate was estimated using the United Nation’s definition. (*21*) The socioeconomic status (SES) of each household was estimated and ranked into rich, intermediate and poor using principal components analysis. (*22*) Variables used in the SES ranking have been described elsewhere. (*23*) Two entomological indices derived from the entomological survey, pupae per household and pupae per person (number of pupae divided by number of persons in each house) were used as potential risk factors for DLI. (*24*) National surveillance system data on dengue incidences in both countries were compared with the DLI data obtained in this study. Comparisons within and between countries were conducted using descriptive analysis. Univariable and multivariable logistic regression models were used to find significant relationships between the presence of DLI and various risk factors in each village. Variables with a significance level of *P* ≤ 0.25 derived from the univariable analysis were included in the multivariable model. A backward stepwise selection procedure was used to obtain significant risk factors (*P* < 0.05) from the multivariable analysis. Statistical analyses were done using STATA (version 10, STATA Corporation, College Station, TX, USA).

### Ethics

All participants and guardians of children signed informed consent forms to participate in the study. The study was approved by the National Ethics Committee for Health Research, Ministry of Health, Vientiane, Lao People's Democratic Republic (No. 03) and by the Ethical Committee of Phramongkutklao College of Medicine, Bangkok, Thailand (S033h/53).

## Results

### Socio-demographic characteristics

Information on the study villages is shown in **Table 1**. The mean ages of people from suburban and rural Lao People's Democratic Republic were 26 and 28 years, while the Thai villagers were older, mean 40 and 42 years, respectively. The main occupation reported was agriculture, especially in the rural villages of both countries, where 65% of individuals were farmers. The population densities in the Lao People's Democratic Republic villages based on the room occupancy were more than 2.5 persons per habitable room, which was higher than the Thai sites. Generally, Thai villages had higher SESs than those in the Lao People's Democratic Republic.

### Entomological survey

Water containers were infested with *Ae. aegypti* pupae in all study villages (**Table 1**). *Aedes aegypti* pupal indices were higher in Thailand than in the Lao People's Democratic Republic; suburban Thailand had the highest numbers of pupae per household (8.7) and pupae per person (2.5). Similar figures were recorded in rural Thailand. With 5.0 pupae per household and 0.9 pupae per person, rural Lao People's Democratic Republic had the lowest *Ae. aegypti* pupal indices recorded in this study.

#### Dengue-like illnesses and confirmed dengue cases

A total of 83 DLI cases were reported during the study period with 69 (mean age: 25 years) in suburban Lao People's Democratic Republic, three in rural Lao People's Democratic Republic (mean age: 49 years) and 11 in rural Thailand (mean age: 42 years). There were no cases recorded in suburban Thailand (**Table 1**). Of the 83 cases, four were confirmed DENV positive (4.8%): two from suburban Lao People's Democratic Republic (both DENV-1) and two from rural Thailand (both DENV-2). Each of these four DENV-positive cases was reported as a DLI case just one time during the study period, and all had sought care at local hospitals. The time from reported date of illness onset to specimen collection was 5 and 9 days, respectively, for the two cases in suburban Lao People's Democratic Republic and 2 and 7, respectively, days for the cases in Thailand.

In suburban Lao People's Democratic Republic, DLI cases were recorded during the entire study period in both 2011 (34 cases) and 2012 (34 cases). The majority of the cases in rural Thailand (10 cases) were recorded in 2011 (**Fig. 2A**). Most of the cases were found during the end of the rainy seasons (August to October in 2011 and October to November in 2012). The confirmed dengue cases were identified around these time periods. In rural Thailand, the confirmed dengue cases were found in November and December 2011.

**Fig. 2A:**
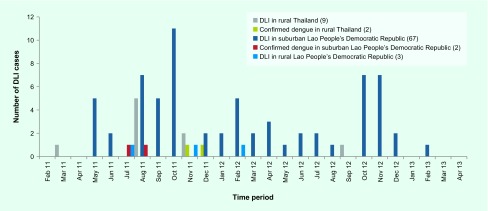
Temporal distribution of dengue-like illness (DLI) and confirmed dengue cases in suburban and rural villages in Lakhonpeng district, Lao People's Democratic Republic (DLI = 72) and Manchakhiri district, Thailand (DLI = 11)

* Figures in parentheses indicate number of cases

Secondary dengue data collected from the Thai national surveillance system provided by the Manchakhiri district hospital surveillance unit showed only five and two confirmed dengue cases in 2011 from the suburban and rural village, respectively. Of these seven cases, only one from the rural village was enrolled in our study and was also confirmed as positive for DENV infection. The other six cases were not in the selected households. In the Lao People's Democratic Republic, no dengue surveillance data at the village level were available.

District-level secondary dengue data obtained from both national surveillance systems showed at least a threefold higher dengue incidence in the Lakhonpheng district (Lao People's Democratic Republic) than in the Manchakhiri district (Thailand) (**Fig. 2B**). In the Lakhonpheng district, the incidence of dengue in 2010 was more than three times higher than in 2011 or 2012 and slightly higher than in 2013. In the Manchakhiri district, dengue incidence was low (< 240 cases/100 000 population) during 2010–2013.

**Fig. 2B:**
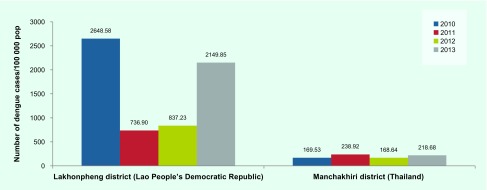
Dengue incidence in Lakhonpheng district, Lao People's Democratic Republic and in Manchakhiri district, Thailand, 2010–2013

Figures represent the number of dengue cases per 100 000 populations reported in the districts in the Lao People's Democratic Republic and in Thailand where the study villages are located (secondary data derived from the national surveillance system).

### Risk factors and dengue-like illnesses

The results from the univariable (**Table 2**) and multivariable (**Table 3**) analyses were similar, and no correlation was found with *Ae. aegypti* pupal indices (**Table 3**). The univariable analysis showed that risk factors associated with DLI in suburban Lao People's Democratic Republic were age, education and occupation. Only age and occupation remained significantly significant in the multivariable analysis. In the 15–20 years age group, the odds of having DLI symptoms were almost five times higher than the odds of those under 5 years of age. The odds of DLI in service and “other” (retired and children) occupations were about three times higher than the odds for farmers. In rural Thailand, the multivariable analysis showed no significant associations between DLI and any risk factor (**Table 3**).

**Table 2:** Univariable analyses of risk factors associated with dengue-like illnesses (DLI) in suburban Lao People's Democratic Republic and in rural Thailand (Odds ratio (OR) ([95% confidence intervals, CI] p-value). Numbers in bold indicate significant associations (*P* < 0.05)

-		Lao People's Democratic Republic – Suburban village (*n* = 567)				Thailand – Rural village (*n* = 402)			
-		%	OR	95% CI	p-value	%	OR	95% CI	p-value
**Socio-demography**									
**Age group**	**0–5**	**13**	**1**	-	-	**7**	**1**	-	-
	** > 5–10**	**10**	**1.6**	**[0.5–4.8]**	**0.390**	**5**	**NA**	-	-
	** > 10–15**	**14**	**2.1**	**[0.8–5.5]**	**0.136**	**5**	**NA**	-	-
	** > 15–20**	**12**	**2.7**	**[1.0–6.9]**	**0.046**	**8**	**NA**	-	-
	** > 20–25**	**6**	**1.0**	**[0.3–4.1]**	**0.970**	**2**	**NA**	-	-
	** > 25**	**45**	**1.3**	**[0.5–3.2]**	**0.560**	**73**	**NA**	-	-
**Sex**	**Male**	**48**	**1**	-	-	**50**	**1**	-	-
	**Female**	**52**	**1.1**	**[0.7–1.8]**	**0.572**	**50**	**4.6**	**[0.9–21.1]**	**0.052**
**Education**	≤ **Primary school**	**53**	**1**	-	-	**77**	**1**	-	-
	** > Primary school**	**47**	**2.3**	**[1.4–3.8]**	**0.001**	**23**	**1.9**	**[0.6–6.5]**	**0.311**
**Occupation**	**Agriculture**	**31**	**1**	-	-	**65**	**1**	-	-
	**Service**	**16**	**2.2**	**[1.0–4.8]**	**0.041**	**3**	**NA**	-	-
	**Commerce**	**6**	**1.3**	**[0.4–4.8]**	**0.643**	**2**	**NA**	-	-
	**Unemployed**	**2**	**3.7**	**[0.8–16.6]**	**0.086**	**2**	**4.5**	**[0.6–36.8]**	**0.158**
	**Student**	**26**	**2.6**	**[1.3–5.2]**	**0.005**	**14**	**1.3**	**[0.3–6.3]**	**0.734**
	Other*	**19**	**1.6**	**[0.7–3.6]**	**0.229**	**14**	**0.7**	**[0.1–5.3]**	**0.694**
**Room occupancy rate**	** > 2.5 persons/room**	**56**	**1**	-	-	**30**	**1**	-	-
	≤ **2.5 persons/room**	**44**	**0.8**	**[0.5–1.3]**	**0.391**	**70**	**1.2**	**[0.3–4.4]**	**0.827**
**Housing material**	**Cement**	**18**	**1**	-	-	**12**	**1**	-	-
	**Cement-wood**	**42**	**1.1**	**[0.6–2.2]**	**0.737**	**63**	**NA**	-	-
	**Wood**	**40**	**0.9**	**[0.5–1.8]**	**0.788**	**25**	**NA**	-	-
**Wealth status**	**Poor**	**29**	**1**	-	-	**14**	**1**	-	-
	**Intermediate**	**42**	**1.3**	**[0.7–2.4]**	**0.375**	**53**	**0.3**	**[0.1–1.1]**	**0.065**
	**Rich**	**29**	**1.5**	**[0.8–2.8]**	**0.227**	**33**	**0.3**	**[0.1–1.5]**	**0.144**
***Aedes aegypti* pupal indices**									
**No. of pupae per household**	-	**100**	**1.0**	**[0.9–1.0]**	**0.917**	**100**	**NA**	-	-
**No. of pupae per person**	**0–0.49**	**67**	**1**	-	-	**51**	**1**	-	-
	**0.5–1.5**	**9**	**0.7**	**[0.2–1.9]**	**0.474**	**21**	**0.6**	**[0.1–2.6]**	**0.450**
	** > 1.5**	**24**	**1.2**	**[0.7–2.0]**	**0.580**	**28**	**NA**	-	-

* Other: retired and children; NA: not applicable

**Table 3:** Multivariable analyses of risk factors associated with dengue-like illnesses (DLI) in suburban Lao People's Democratic Republic and rural Thailand (Odds ratio (OR) ([95% confidence intervals, CI] p-value). Numbers in bold indicate significant associations (*P* < 0.05)

-		Lao People's Democratic Republic – Suburban village (*n* = 567)				Thailand – Rural village (*n* = 402)			
-		%	OR	95% CI	p-value	%	OR	95% CI	p-value
**Age group**	**0–5**	**13**	**1**	-	-	**7**	**1**	-	-
	** > 5–10**	**10**	**2.1**	**[0.6–6.9]**	**0.251**	**5**	**NA**	-	-
	** > 10–15**	**14**	**3.4**	**[0.8–14.8]**	**0.097**	**5**	**NA**	-	-
	** > 15–20**	**12**	**4.8**	**[1.2–19.6]**	**0.029**	**8**	**NA**	-	-
	** > 20–25**	**6**	**2.1**	**[0.4–11.9]**	**0.406**	**2**	**NA**	-	-
	** > 25**	**45**	**2.8**	**[0.8–10.5]**	**0.122**	**73**	**NA**	-	-
**Sex**	**Male**	-	-	-	-	**50**	**1**	-	-
	**Female**	-	-	-	-	**50**	**4.2**	**[0.9–19.3]**	**0.068**
**Occupation**	**Agriculture**	**31**	**1**	-	-	-	-	-	-
	**Service**	**16**	**2.4**	**[1.1–5.4]**	**0.028**	-	-	-	-
	**Commerce**	**6**	**1.4**	**[0.4–5.0]**	**0.606**	-	-	-	-
	**Unemployed**	**2**	**3.2**	**[0.7–14.7]**	**0.136**	-	-	-	-
	**Student**	**26**	**2.3**	**[0.8–6.1]**	**0.104**	-	-	-	-
	Other*	**19**	**3.5**	**[1.1–11.0]**	**0.031**	-	-	-	-
**Wealth status**	**Poor**	-	-	-	-	**14**	**1**	-	-
	**Intermediate**	-	-	-	-	**53**	**0.3**	**[0.1–1.2]**	**0.081**
	**Rich**	-	-	-	-	**33**	**0.3**	**[0.1–1.4]**	**0.129**

* Other: retired and children; NA: not applicable

Several methods for dengue protection were used by DLI cases (**Table 1**): indoor aerosol insecticide spray, mosquito coils, repellents, etc.; however, they were rarely recorded. Mosquito nets were thought to be protective, and they were used by 100% of cases in suburban and rural Lao People's Democratic Republic and by 73% of cases in rural Thailand.

## Discussion

### Dengue and dengue-like illnesses

Eighty-three DLI cases were recorded among 2007 inhabitants during the two-year study period (**Table 1**). Only one case, recorded in suburban Lao People's Democratic Republic, was from a participant newly enrolled during the study period. Most of the cases were recorded during the rainy season, consistent with previous findings. (*1*, *4*) The incidence of DLI in the Lao People's Democratic Republic was two times higher than in Thailand, which corresponds with the district-level dengue surveillance data reported from the same study periods in both countries (**Fig. 2B**). We also found that dengue incidence in Lakhonpheng district (Lao People's Democratic Republic) was three to four times higher than in Manchakhiri district (Thailand).

No DLI was reported in suburban Thailand, although the national surveillance system reported five dengue cases from this village. The affected households were not included in our study. Fewer DLIs were recorded in rural Lao People's Democratic Republic (three in total). One factor that could contribute is that the study village has no health facility; therefore, people may not seek health care.

Only four of the 83 DLI cases (4.8%) were confirmed as dengue in this study. Non-confirmed DLIs could have resulted from false negative dengue or from other diseases that present with similar clinical manifestations. (*9*) A study of a cohort of 1500 healthy children aged 2–14 years in Indonesia, Malaysia, the Philippines, Thailand, and Viet Nam found that the most common causes of AFI (≥ 38 °C for ≥ 2 days) were chikungunya, scrub typhus, and dengue. (*25*) Co-circulating arboviruses, such as Zika and chikungunya, pose challenges for disease diagnosis and early response to outbreaks (*26*) since they are often indistinguishable clinically. (*27*, *28*) In South-East Asia, Zika virus was first reported in Malaysia in 1966, and subsequent cases were also reported in many countries including in Thailand (2014). (*29*) Recently, a previous Zika outbreak in the region was recorded in Singapore (2016). (*28*) In the Lao People's Democratic Republic and in Thailand, the first chikungunya outbreaks were reported in 1958 (*30*) and 2012, (*31*) respectively. Other infections that cause DLIs in the study region include scrub typhus, influenza, Japanese encephalitis and leptospirosis. (*16*, *32*)

The unexpectedly low number of confirmed dengue infections could be due to sample degradation from inadequate temperature control from intermittent power supplies or the use of only one laboratory method to detect infections. Although real-time PCR testing has a reported sensitivity of 93%, (*20*) some samples could have been false negatives. Using both viral detection and serological tests, such as IgM ELISA, would improve diagnostic accuracy.

This study revealed circulation of DENV-1 in the Lao People's Democratic Republic and DENV-2 in Thailand, a finding corroborated by national surveillance data. DENV-1 was detected in the Lao People's Democratic Republic during 2007–2011 and accounted for the highest proportion of dengue serotypes (38%) during the 2010 outbreak, followed by DENV-2 (30%). (*1*) In Thailand, 54.6% of the DENV serotypes isolated in 2010 were DENV-2, followed by DENV-1 (25.5%). (*4*)

### Risk factors of dengue-like illnesses

Significant risk factors for DLI were found only in the suburban village of the Lao People's Democratic Republic, where DLI was associated with age and occupation. Individuals aged 15–20 years old were more likely to have DLI than those 0–5 years old, which is in line with a previous study conducted in Brazil. (*33*) In the 2010 dengue outbreak in the Lao People's Democratic Republic, the most affected age group was 10–19 years, (*1*) similar to the findings of this study. In Thailand, the highest incidence rates of dengue reported between 2000 and 2011 were in 10–14 year olds. (*4*) Similar findings occurred during a 2009 chikungunya outbreak in Thailand where the most affected age group was 10–14 years. (*34*)

Employment in a service occupation was associated with DLI (**Table 3**), and 80% of these employees had an educational level higher than high school. A previous study found that attainment of secondary or higher educational degrees was significantly associated with dengue infection. (*35*) This may relate to travel or work patterns away from home, thus increasing their chances of contracting dengue infections compared with those who travel less. Human movement as a result of socieconomic development favours the spread of dengue and other vector-borne diseases. (*36*, *37*)

Clustering of DLI cases could be influenced by household risk within the same household; however, a household-level spatial analysis of DLI cases was not conducted in this study. Furthermore, dengue transmission is not limited to within households. Schools, workplaces, markets, hospitals, parks, and other public places may play a role in dengue transmission. Dengue control interventions that focus on households may be insufficient for community-wide disease control.

Although pupal indices are accepted as better indicators of dengue transmission than the traditional *Stegomyia* indices (i.e. House, Container, and Breteau indices), (*38*–*40*) a high density (> 1.5 pupae per person) of *Ae. aegypti* was not associated with DLI in the household (**Table 2**), even though the pupal densities found in three of the four study villages (**Table 1**) were above proposed transmission thresholds of 0.5–1.5 pupae per person. (*24*) Similarly, a study conducted in the Republic of Palau found that DLI infections were not associated with the pupal index; however, households reporting DLIs were significantly more likely to harbour potential mosquito breeding sites than those without. (*15*) A systematic review of the correlation between vector indices and dengue transmission also found no robust relationships to predict dengue outbreaks. (*40*) More reliable mosquito indices are needed. Adult mosquito collections may provide more useful information of disease risk, since infectious adult mosquitoes are more epidemiologically relevant than larvae or pupae. (*9*)

Although our study focused only on dengue, other febrile illnesses, such as chikungunya and Zika, are also endemic in these locations. Our findings corroborate those from the national dengue surveillance programmes, highlighting the importance of continued clinical and vector surveillance and indicating the need to expand surveillance to include other mosquito-borne diseases associated with AFI. This would have significant impact on accurate and timely detection and reporting AFI-related outbreaks.
